# Sexually dimorphic murine brain uptake of the 18 kDa translocator protein PET radiotracer [^18^F]LW223

**DOI:** 10.1093/braincomms/fcae008

**Published:** 2024-01-16

**Authors:** Agne Knyzeliene, Catriona Wimberley, Mark G MacAskill, Carlos J Alcaide-Corral, Timaeus E F Morgan, Martyn C Henry, Christophe Lucatelli, Sally L Pimlott, Andrew Sutherland, Adriana A S Tavares

**Affiliations:** British Heart Foundation-University of Edinburgh Centre for Cardiovascular Science, University of Edinburgh, Edinburgh EH16 4TJ, UK; Edinburgh Imaging, University of Edinburgh, Edinburgh EH16 4TJ, UK; Edinburgh Imaging, University of Edinburgh, Edinburgh EH16 4TJ, UK; Centre for Clinical Brain Sciences, University of Edinburgh, Edinburgh EH16 4SB, UK; British Heart Foundation-University of Edinburgh Centre for Cardiovascular Science, University of Edinburgh, Edinburgh EH16 4TJ, UK; Edinburgh Imaging, University of Edinburgh, Edinburgh EH16 4TJ, UK; British Heart Foundation-University of Edinburgh Centre for Cardiovascular Science, University of Edinburgh, Edinburgh EH16 4TJ, UK; Edinburgh Imaging, University of Edinburgh, Edinburgh EH16 4TJ, UK; British Heart Foundation-University of Edinburgh Centre for Cardiovascular Science, University of Edinburgh, Edinburgh EH16 4TJ, UK; Edinburgh Imaging, University of Edinburgh, Edinburgh EH16 4TJ, UK; School of Chemistry, University of Glasgow, Glasgow G12 8QQ, UK; Edinburgh Imaging, University of Edinburgh, Edinburgh EH16 4TJ, UK; West of Scotland PET Centre, Greater Glasgow and Clyde NHS Trust, Glasgow G12 0YN, UK; School of Chemistry, University of Glasgow, Glasgow G12 8QQ, UK; British Heart Foundation-University of Edinburgh Centre for Cardiovascular Science, University of Edinburgh, Edinburgh EH16 4TJ, UK; Edinburgh Imaging, University of Edinburgh, Edinburgh EH16 4TJ, UK

**Keywords:** TSPO, PET, [^18^F]LW223, sex differences

## Abstract

The 18 kDa translocator protein is a well-known biomarker of neuroinflammation, but also plays a role in homeostasis. PET with 18 kDa translocator protein radiotracers [^11^C]PBR28 in humans and [^18^F]GE180 in mice has demonstrated sex-dependent uptake patterns in the healthy brain, suggesting sex-dependent 18 kDa translocator protein expression, although humans and mice had differing results. This study aimed to assess whether the 18 kDa translocator protein PET radiotracer [^18^F]LW223 exhibited sexually dimorphic uptake in healthy murine brain and peripheral organs. Male and female C57Bl6/J mice (13.6 ± 5.4 weeks, 26.8 ± 5.4 g, mean ± SD) underwent 2 h PET scanning post-administration of [^18^F]LW223 (6.7 ± 3.6 MBq). Volume of interest and parametric analyses were performed using standard uptake values (90–120 min). Statistical differences were assessed by unpaired *t*-test or two-way ANOVA with Šidak’s test (alpha = 0.05). The uptake of [^18^F]LW223 was significantly higher across multiple regions of the male mouse brain, with the most pronounced difference detected in hypothalamus (*P* < 0.0001). Males also exhibited significantly higher [^18^F]LW223 uptake in the heart when compared to females (*P* = 0.0107). Data support previous findings on sexually dimorphic 18 kDa translocator protein radiotracer uptake patterns in mice and highlight the need to conduct sex-controlled comparisons in 18 kDa translocator protein PET imaging studies.

## Teaser

Knyzeliene *et al.* report sexually dimorphic murine brain and heart uptake of the novel 18 kDa translocator protein (TSPO) PET radiotracer [^18^F]LW223. Results confirm that [^18^F]LW223 can detect changes in TSPO *in vivo* and with high sensitivity.

## Graphical Abstract

**Graphical Abstract:**
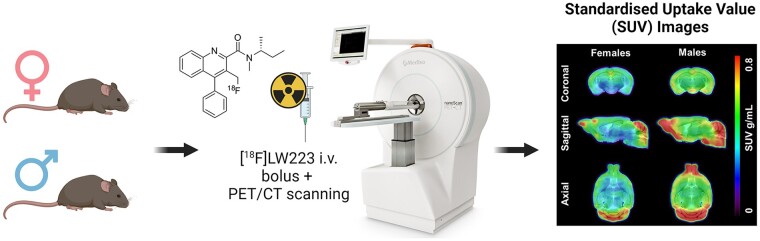

## Introduction

The link between sex and susceptibility to a number of neurological diseases and disorders has been well defined and new evidence is constantly emerging.^1,2^ However, prior to studying disease states, and especially when characterizing disease biomarkers, it is important to understand whether any sex-dependent differences are present under healthy conditions. Recent PET imaging studies identified such differences for the 18 kDa translocator protein (TSPO), which is usually considered a biomarker for inflammation, but is also known to play a role in steroidogenesis, mitochondrial energy metabolism, cell proliferation, apoptosis and immunomodulation.^3-6^ Tuisku *et al*.^7^ have shown that in human subjects, binding of the PET radiotracer [^11^C]PBR28 to TSPO in the brain was 16.3% higher in females than in males. They also found that with age, [^11^C]PBR28 binding to TSPO in the brain increased in male, but not in female subjects. Conversely, a preclinical study by Biechele *et al*.^8^ showed that male mice had a higher brain uptake of another TSPO PET radiotracer, [^18^F]GE180, than females. This study also demonstrated that with age, [^18^F]GE180 uptake in the murine male brain remained stable, whereas it increased in females. In young adult wild-type mice, [^18^F]GE180 brain uptake was ∼10–15% higher in males compared with females.^8^ These early findings suggested that cross-gender, cross-species and cross-radiotracer binding differences may exist among PET radiotracers targeting TSPO, highlighting the need to carefully investigate these aspects for current and new compounds.

The current study aimed to assess sex differences in the uptake of the TSPO PET radiotracer [^18^F]LW223 in healthy mice, hypothesizing that higher brain uptake would be observed in males compared with females (based on previous results in mice).^8^ Given [^18^F]LW223 unique *in vivo* properties, including low non-displaceable volume (*V*_ND_) in mouse brain, we also hypothesized that our novel radiotracer had the potential to detect greater differences between males and females with potential to unravel new biological insights by enabling higher sensitivity analysis of regional TSPO changes in the murine brain versus other previously developed TSPO PET radiotracers.^9^ Having rs6971 polymorphism-independent binding in human tissue *ex vivo*, [^18^F]LW223 holds great potential to enable TSPO PET imaging across the whole human population.^10,11^ Previously, we have also shown that [^18^F]LW223 has excellent properties as an imaging biomarker, including low radiometabolism, high free fraction and binding kinetics amenable to the use of simplified outcome measures in both rats and mice.^9,11^ Therefore, prior to its widespread application to image various diseases and disease models, it is important to understand binding patterns of [^18^F]LW223 in healthy males and females. Although this study mainly focussed on differences in the brain, it also assessed radiotracer uptake differences at the whole-body level in mice.

## Materials and methods

### Radiosynthesis of [^18^F]LW223

The structure and radiosynthesis of [^18^F]LW223 were as described previously,^11^ except the mobile phase flow rate used for purification of the final product using a semi-preparative high performance liquid chromatography system was reduced from 5 to 3 mL/min. [^18^F]LW223 was produced with good molar activity, as previously described,^11^ which is compliant with the radiotracer principle, as demonstrated in our previously published mass effect study in mice.^9^

### Animals

The animals used in the study were purchased from Charles River Laboratories (Tranent, UK). All animal experiments were conducted with authorization from the local University of Edinburgh animal welfare and ethical review committee and in accordance with the Home Office Animals (Scientific Procedures) Act 1986. The animals were housed in individually ventilated cages under standard 12 h light:12 h dark conditions with food and water available *ad libitum*. All animals were scanned during the 12 h light cycle conditions.

### [^18^F]LW223 PET imaging

Young male (*n* = 9, age = 14.69 ± 6.15 weeks, weight = 29.12 ±3.27 g) and female (*n* = 5, age = 11.56 ± 2.45 weeks, weight =22.56 ± 5.77 g) C57Bl/6J mice were used in the PET study. The data of seven out of the nine male and five female mice were repurposed from our previous study by MacAskill *et al*.^11^ Animals were anaesthetized using 1.5–2% isoflurane (Isoflo® APIECE, Zoetis, UK) (50/50 oxygen/nitrous oxide, 1 L/min) and body temperature was maintained using a heated mat. Tail vein cannulations for injection of [^18^F]LW223 were performed using butterfly needles (27G 1/2″, 12 cm polyurethane tubing, SAI Infusion Technologies, USA), except for two male animals that underwent femoral vein and artery cannulation for radiotracer injection and blood sampling.

PET scans were performed immediately post intravenous bolus injection of [^18^F]LW223 (*n* = 14, 6.36 ± 3.70 MBq, bolus i.v., mean ± SD). Imaging data were acquired using a preclinical PET/CT scanner (nanoPET/CT, Mediso, Hungary). Respiration rate and body temperature were monitored and maintained throughout the imaging session. A 2 h emission dataset per animal was reconstructed using three-dimensional 1:5 mode and re-binned as follows: 18 × 10 s, 2 × 30 s, 1 × 60 s, 2 × 120 s, 10 × 300 s and 6 × 600 s. All PET studies were reconstructed using Mediso’s iterative Tera-Tomo 3D reconstruction algorithm, which includes point spread correction, and the following settings: four iterations, six subsets, full detector model, normal regularization, spike filter on, voxel size of 0.2 mm and 400–600 keV energy window. Corrections for randoms, scatter and attenuation were applied to all PET data. Immediately post PET imaging session, a 5 min CT scan (semi-circular full trajectory, maximum field of view, 480 projections, 50 kVp, 300 ms and 1:4 binning) was acquired for attenuation correction and anatomical information. The following parameters were used for CT image reconstruction: matrix size = 121 × 121 × 121 mm, voxel size = 0.25 × 0.25 × 0.25 mm, cosine filter, cut-off at 100%, corrections for offset, gain and pixel.

### PET image processing and analysis

Reconstructed images were analysed using PMOD version 3.7 (PMOD Technologies, Switzerland). Volumes of interest (VOIs) were manually drawn around the whole brain, whole heart and whole left and right lungs (with both lungs merged afterwards) using CT images. The whole organ VOIs of spleen, kidneys, liver, adrenals and eyes were drawn using averaged PET images (0–120 min). For regional brain analysis, PET data were co-registered with the mouse brain T2 MRI template and the modified Mirrione mouse brain atlas was used to generate VOIs of the following brain regions: cortex, thalamus, cerebellum, basal forebrain septum, hypothalamus, brain stem, central grey matter, olfactory bulb, amygdala, midbrain, third ventricle, corpus callosum, striatum and hippocampus.^12^ The data were extracted as time–activity curves and standardized uptake values (SUVs) were calculated as concentration in the VOI divided by the injected dose divided by the animal weight. Average SUV values between 90 and 120 min (SUV_90–120 min_) were used as an outcome measure. This outcome measure was previously validated versus gold-standard invasive kinetic modelling of [^18^F]LW223 murine brain PET datasets.^9^

### Generation of representative PET images

Representative brain and whole-body PET SUV_90–120 min_ images for presentation purposes were generated using PMOD version 3.7 (PMOD Technologies, Switzerland). For brain PET images, Gaussian 3D 1.2 mm filter was applied, and they were co-registered with a mouse brain T2 MRI template, whereas Gaussian 3D 1 mm filter was used for whole-body PET images.

### Parametric brain analysis

To create parametric maps of [^18^F]LW223 uptake in the mouse brain, average male (*n* = 9) and female (*n* = 5) SUV brain maps were generated. For each animal, the SUV was calculated for each voxel of an average image of 90–120 min post-radiotracer injection as in the VOI analysis detailed above. The SUV PET images were normalized to the same space as follows: (i) the CT scans were cropped around the skull and each one was registered to an average MRI atlas by Dorr *et al*.^13^; and (ii) the transformation matrices between the PET, CT and MRI atlas were combined to position each SUV image into the MRI space, therefore aligning all SUV images. Each SUV image was smoothed with a Gaussian kernel of 0.2 × 0.2 × 0.2 mm and the brain was masked using the registered MRI atlas. Three separate average SUV brain maps were created and made freely available online (https://doi.org/10.7488/ds/2988): (i) for male animals, (ii) for female animals and (iii) for all animals, where an average over the animals in each group was calculated for each voxel within the brain. Statistical analysis to determine any significant difference between the male and female groups was carried out using R statistical package and the RMINC (R studio package for Medical Imaging NetCDF (Network Common Data Format)) module. The *t*-statistics calculated between groups were overlaid on the MRI atlas.

### Statistical analysis

Plotting of graphs and statistical analysis was performed using Prism 9.3.1 (GraphPad, USA). Unpaired *t*-test or two-way ANOVA with Šidak’s *post hoc* test (alpha = 0.05) was used as indicated in the relevant figure legends.

## Results

### [^18^F]LW223 uptake was higher in specific regions of the healthy male versus the healthy female murine brain

**Figure 1:**
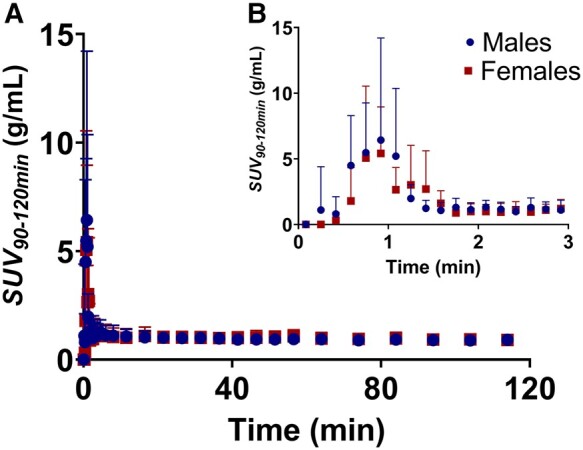
**[^18^F]LW223 whole-blood time–activity curves.** (**A**) Male and female mouse image-derived whole-blood time–activity curves from 0 to 120 min post-radiotracer administration. (**B**) Insert showing whole-blood time–activity curves from 0 to 3 min post-radiotracer administration. Data presented as mean ± SD, *n* = 9 males and *n* = 5 females. SUV, standardized uptake value.

**Figure 2:**
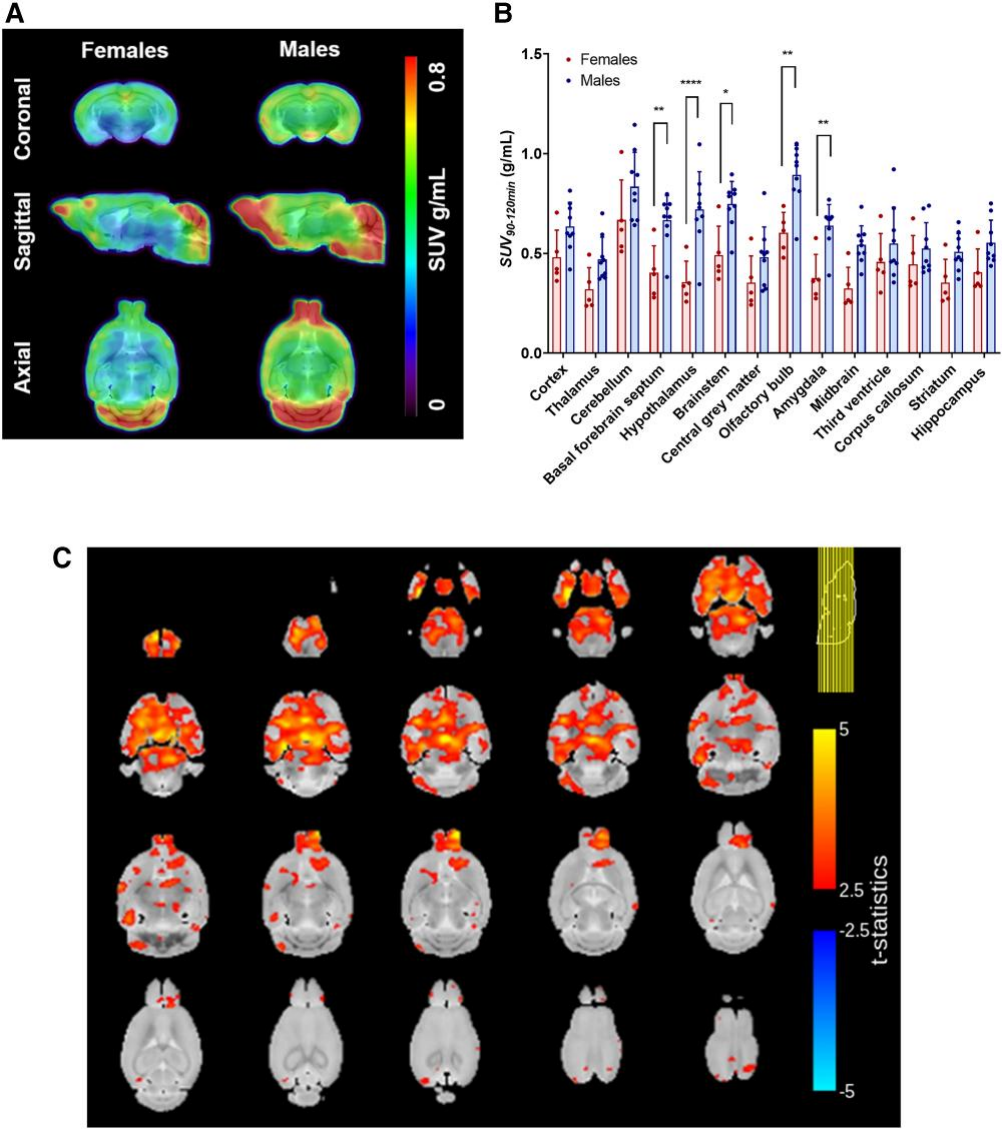
**Sex-dependent [^18^F]LW223 uptake differences detected during regional analysis of a mouse brain.** (**A**) Representative images of female (*n* = 5) and male (*n* = 9) average [^18^F]LW223 standardized uptake values averaged between 90 and 120 min (SUV_90–120 min_) brain atlases. (**B**) A comparison of regional [^18^F]LW223 uptake in female (*n* = 5) and male (*n* = 9) mouse brains (mean ± SD; two-way ANOVA, Šidak’s multiple comparison test, alpha = 0.05, *P = 0.0112, **P ≤ 0.0079, ****P < 0.0001). (**C**) Statistical parametric map showing *t*-statistics for differences between the female (*n* = 5) and male (*n* = 9) brains [^18^F]LW223 SUV_90–120 min_.

[^18^F]LW223 blood uptake and kinetics were comparable in male versus female adult mice (Fig. 1). A 3D TSPO atlas of the female and male mouse brains was generated using SUV_90–120 min_ PET data collected following injection of [^18^F]LW223 (Fig. 2A). The VOI analysis showed that in male mice, [^18^F]LW223 PET uptake was up to 50% higher in the basal forebrain septum, hypothalamus, brain stem, olfactory bulb and amygdala when compared to females (Fig. 2B). This was confirmed by statistical parametric mapping analysis (Fig. 2C), with additional significant differences detected in the striatum, hippocampus, thalamus, midbrain, cerebellum and in the frontal, entorhinal and parieto-temporal cortices (Fig. 2C).

### Sex differences of [^18^F]LW223 uptake identified in the whole brain and heart of healthy mice

**Figure 3:**
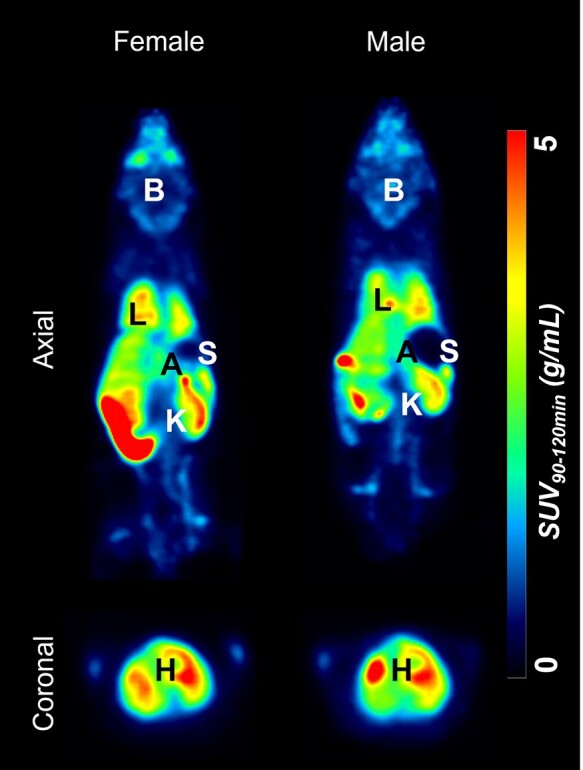
**Representative [^18^F]LW223 standardized uptake values averaged between 90 and 120 min (SUV_90–120 min_) total-body images of female and male mice.** B, brain; L, lungs; H, heart; K, kidneys; A, adrenals; S, spleen.

**Figure 4:**
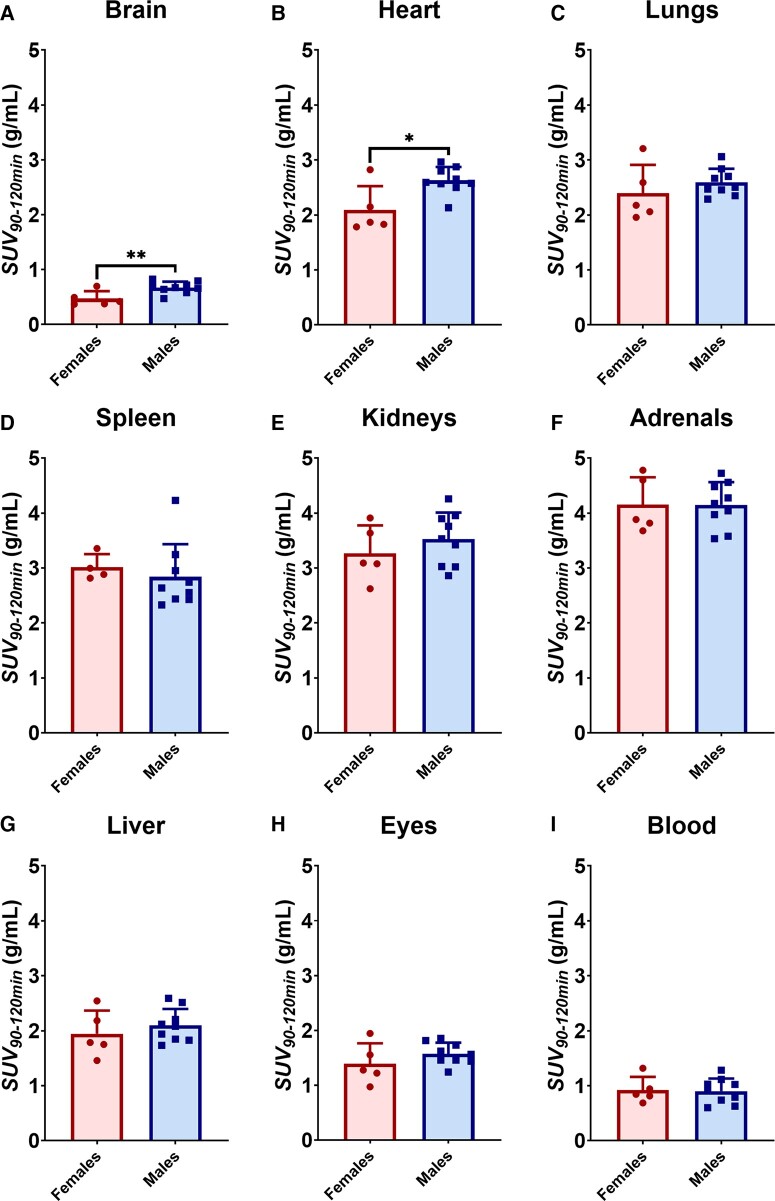
**Young male mice have higher [^18^F]LW223 uptake in brain and heart compared with young female mice.** The data represent uptake of [^18^F]LW223 in brain (**A**), peripheral organs (**B–H**) and blood (**I**) of female (*n* = 5) and male (*n* = 9) mice (mean ± SD; *P = 0.0107, **P = 0.0095, unpaired *t*-test, alpha = 0.05). SUV, standardized uptake value.

In addition to assessing regional uptake of [^18^F]LW223 in male and female mouse brains, SUV_90–120 min_ analysis was performed in peripheral organs that are known to express TSPO (Figs 3 and 4). It was found that in addition to having significantly higher SUV_90–120 min_ in the brain of healthy males (42% difference), the uptake of [^18^F]LW223 was also significantly higher in the heart of male mice (Fig. 4). Other organs, such as the lungs, spleen, kidneys and adrenals, did not present a sex-dependent SUV_90–120 min_ of [^18^F]LW223.

## Discussion

Sexual dimorphism in protein expression is becoming increasingly acknowledged in the field of neuroscience. For decades, the majority of neuroscience research has been performed predominately in males, disregarding the fact that the expression of disease markers may significantly differ between males and females even under healthy conditions, leading to knowledge gaps across the field.^14^ Recently published data suggested that TSPO may also be one of the proteins within this category.^7,8^

Although the present study did not assess the effect of aging on [^18^F]LW223 binding in male and female mouse brains, the results paralleled the outcomes of the equivalently aged C57Bl/6 mice from the Biechele *et al*. study, where young males had significantly higher [^18^F]LW223 SUV_90–120 min_ across multiple brain regions when compared to females, supporting our hypothesis. Results from this study also showed that global (42%) and regional (up to 50%) changes in the [^18^F]LW223 murine male brain uptake versus the female murine brain were more striking than previous reports using other TSPO PET radiotracers, namely [^18^F]GE180 (10–15% difference). This confirms our secondary hypothesis that [^18^F]LW223 has superb sensitivity for detection of TSPO changes in the murine brain and reflects the lower *V*_ND_ of [^18^F]LW223. Furthermore, we have previously shown that [^18^F]LW223 has good brain penetration followed by slow clearance due to its high affinity to murine TSPO.^9^

The reasons behind sexual dimorphism of TSPO are still to be investigated, but a working hypothesis is that it may be involved in sex-dependent regulation of neurosteroid production in the brain. This would resemble expression patterns of other proteins involved in neurosteroidogenesis, such as steroidogenic acute regulatory protein (StAR), 5 alpha reductase (5α-R) and 3 alpha-hydroxysteroid oxidoreductase (3α-HSOR), which have been found to differ between male and female rodents.^15^ Moreover, levels of neurosteroids, such as dihydroprogesterone, tetrahydroprogesterone, isopregnanolone, dehydroepiandrosterone, testosterone and others, also show regional differences in distribution between male and female brains.^16^ However, it is still unclear why rodents and humans exhibit opposite trends when it comes to sexual dimorphism of TSPO.^7^ Our results presented here confirm and expand prior observations with a different TSPO radiotracer [^18^F]GE180 in mice.

The uptake of [^18^F]LW223 in peripheral mouse organs with known TSPO expression was also investigated. It is important to gain this understanding as TSPO PET imaging is increasingly applied for imaging inflammation beyond the brain, as well as for assessing functional heart–brain TSPO axis.^11,17,18^ Opposed to previous findings on relative TSPO gene expression in Balb/cJ mice, in the current study, C57Bl/6J males exhibited significantly higher uptake of [^18^F]LW223 in the heart when compared to females, indicating higher levels of TSPO.^19^ Although this contradictory finding could be influenced by gene expression differences between Balb/c and C57Bl/6 mouse strains, these results highlight the importance of conducting sex-controlled TSPO PET imaging studies when assessing functional TSPO axes or inflammatory conditions across the body.^20^

Since SUV_90–120 min_ was performed to quantify [^18^F]LW223 PET data in this study, it is possible that the results might be impacted by possible confounding factors, such as sex-dependent cerebral blood flow (CBF) differences. However, no data are currently available to support this hypothesis, as no sex-dependent differences in CBF were previously found in rodents.^21^

As eluded previously, [^18^F]LW223 has a number of important advantages compared with previously developed TSPO radiotracers, which can resolve the current bottleneck in clinical TSPO PET imaging. Here, we show that [^18^F]LW223 uptake across mouse brain regions and heart is sex-dependent, with male mice expressing higher levels of TSPO when compared to females. Gaining this information during the preclinical development of [^18^F]LW223 is beneficial, as it aids better design of future preclinical and clinical studies with this lead TSPO radiotracer, for which a translational package is already available, including dosimetry analysis.^11^ Our findings also highlighted the need for studies investigating sexually dimorphic target expression patterns across the field of preclinical neuroscience, which would expand the current understanding on brain functions and allow for the use of patient-tailored diagnostic measures.

## Data Availability

Upon manuscript acceptance, all study data will be deposited in the ‘PET is Wonderful’ collection, hosted at the University of Edinburgh DataShare platform: https://datashare.ed.ac.uk/handle/10283/3219.
